# Use of a three-band HRP2/pLDH combination rapid diagnostic test increases diagnostic specificity for falciparum malaria in Ugandan children

**DOI:** 10.1186/1475-2875-13-43

**Published:** 2014-02-01

**Authors:** Michael Hawkes, Andrea L Conroy, Robert O Opoka, Sophie Namasopo, W Conrad Liles, Chandy C John, Kevin C Kain

**Affiliations:** 1 Division of Pediatric Infectious Diseases, Department of Pediatrics, University of Alberta, Edmonton, Canada; 2 Department of Medicine, University of Toronto, Toronto, Canada; 3 Department of Paediatrics and Child Health, Mulago Hospital and Makerere University, Kampala, Uganda; 4 Department of Paediatrics, Jinja Regional Referral Hospital, Jinja, Uganda; 5 Department of Medicine, University of Washington, Seattle, WA, USA; 6 Division of Global Pediatrics, Department of Pediatrics, University of Minnesota, Minnesota, USA; 7 Institute of Medical Sciences, University of Toronto, Toronto, Canada; 8 SAR Laboratories, Sandra Rotman Centre for Global Health, Toronto, Canada; 9 Tropical Disease Unit, University Health Network-Toronto General Hospital, Toronto, Canada

**Keywords:** Malaria, Rapid diagnostic test, Sensitivity, Specificity

## Abstract

**Background:**

Rapid diagnostic tests (RDTs) for malaria provide a practical alternative to light microscopy for malaria diagnosis in resource-limited settings. Three-band RDTs incorporating two parasite antigens may have enhanced diagnostic specificity, relative to two-band RDTs with a single parasite antigen (typically histidine-rich protein 2 [HRP2]).

**Methods:**

Phase 1: 2,000 children, two months to five years of age, admitted to a referral hospital in Jinja, Uganda, with acute febrile illness were enrolled. A WHO highly rated three-band RDT was compared to light microscopy of thick peripheral blood films read by local expert microscopists.

Phase 2: the three-band RDT was used as a screening tool for inclusion of patients in a clinical trial, and subjects with three positive RDT bands were tested by microscopy using blood samples drawn in parallel. Discordant results were adjudicated by PCR.

**Results:**

Phase 1: 1,648 children had both a RDT and peripheral blood smear performed. The specificity of a RDT with all three bands positive was 82% (95% CI: 79-85%) compared to 62% (95% CI: 59-66%) for HRP2 alone. The sensitivity was 88% (95% CI: 85-89%) and 94% (95% CI: 92-95%) for three-band positive RDT and HRP2 antigen, respectively. 119 patients (7.2%) had a positive HRP2 band, but negative parasite lactate dehydrogenase (pLHD) band and negative peripheral smear, and 72 (61%) of these had received pre-treatment with anti-malarials, suggesting a false positive HRP2 result (p = 0.002).

Phase 2: the positive predictive value (PPV) of the three-band RDT was 94% (95% CI 89%-97%) using microscopy as the reference standard. However, microscopy-discordant results were shown to be positive for *P. falciparum* by PCR in all cases, suggesting that the PPV was in fact higher.

**Conclusion:**

The pLDH antigen on three-band RDTs, used in combination with HRP2, provides added diagnostic specificity for malaria parasitaemia and may be useful to distinguish acute infection from recently treated infection. In situations where diagnostic specificity is desirable (e.g., for selection of malaria-infected participants in clinical trials), a three-band RDT should be considered in a sub-Saharan African setting.

## Background

Immunochromatographic rapid diagnostic tests (RDTs) are practical and accurate tools to guide case management of clinical malaria, particularly in resource-constrained settings where routine microscopy is absent or of poor quality. In addition to their clinical utility, RDTs have been used for epidemiologic surveillance [1,2], and for selection of patients for clinical trials of new therapies [3,4]. The test performance characteristics of RDTs have been described in numerous reports from Asia and Africa for the purpose of malaria case management, where high sensitivity is desirable in order to avoid false negative results that could lead to inappropriately withholding treatment from patients with malaria [5,6]. In other applications, such as selection of patients for clinical trials, test specificity may be paramount, in order to avoid false positive tests which would lead to inappropriate inclusion of patients without malaria.

The test principle underlying malaria RDTs is the detection of parasite antigens, most commonly histidine-rich protein 2 (HRP2), parasite lactate dehydrogenase (pLDH), and/or parasite aldolase through lateral flow immunochromatography. Whereas HRP2 is specific to *Plasmodium falciparum*, pLDH and parasite aldolase are common to all Plasmodium species. Test cartridges or strips may incorporate two bands (e.g., HRP2 and a control band), or three bands (e.g., HRP2, pLDH, and a control band). The use of three-band RDTs allows the distinction of *P. falciparum* from *Plasmodium vivax* in settings, such as Asia and South America, where both species co-circulate [7]. On the other hand, in sub-Saharan Africa, the predominant pathogen is *P. falciparum*, and two-band RDTs incorporating HRP2 antigen as the sole antigen are sensitive tests for malaria diagnosis in clinical practice [8]. One well-recognized limitation of two-band RDTs is the occurrence of false-positive results caused by persistent HRP2 antigenaemia after effective anti-malarial treatment [9,10]. In epidemiologic surveillance, this may result in over-estimation of the true parasite prevalence by 1.5 to 7.9% [11,12]. For recruitment of participants into clinical trials of new treatments, highly specific diagnosis with rapid turn-around time is desirable in order to accurately identify participants that may benefit from the experimental therapy while minimizing enrolment of uninfected participants, who may be unnecessarily exposed to study risks and contaminate study analyses.

The objective of this study was to examine whether a three-band RDT, combining both HPR2 and pLDH antigens, would have increased diagnostic specificity for the diagnosis of *P. falciparum* in an African context. Test performance characteristics of this three-band RDT were examined in a large cohort of children hospitalized with febrile illness in Africa. Furthermore, this approach was validated using the three-band RDT as a screening tool for patient inclusion in a randomized controlled trial of a novel adjunctive treatment for severe malaria [13].

## Methods

This study involved two phases: the first was a prospective cross-sectional comparison of a three band RDT with microscopy for the diagnosis of *P. falciparum* and the second was validation of the three-band RDT for recruitment of patients with falciparum malaria in the context of a randomized clinical trial [13]. The commercially available RDT used in both phases of this study (First Response Malaria Ag. (pLDH/HRP2) Combo Rapid Diagnostic Test, Premier Medical Corporation Limited, India) is the highest-ranked assay by a standardized WHO testing methodology [14]. Previous reports of its use in India [7] and Yemen [15] support its high sensitivity and specificity.

For the first phase, febrile children presenting to Jinja Regional Referral Hospital, Uganda, were enrolled. Inclusion criteria were: age 2 months to 5 years; parental report of fever within the past 48 hours or axillary temperature higher than 37.5°C; and hospitalization indicated based on the admitting physician’s judgment. Patients were excluded if the accompanying parent or guardian did not provide written informed consent. Demographic and clinical information was collected, and a peripheral blood sample was drawn for testing by both RDT and microscopy. Study personnel consisting of experienced paediatric nurses and medical officers, were trained in the safe use and interpretation of the RDT from a small volume (15 μL) of capillary or venous blood. A simultaneously collected EDTA-anticoagulated sample was spread as a thick smear on a glass slide, dried, stained with Field’s stain, and examined by an experienced technician using a light microscope using a single reading at the Jinja Regional Referral Hospital (paediatric campus). The clinical personnel were not aware of the microscopy result at the time of RDT interpretation, and technician performing the microscopy was not aware of the RDT result. Test performance characteristics (sensitivity, specificity, positive predictive value, negative predictive value) of the RDT were computed, using microscopy as the reference standard. A sample size calculation using standard formulae for the confidence interval on a proportion (assuming sensitivity = specificity = 90%, and disease prevalence in the screened population = 60%) indicated that approximately 1,400 patients would be required to estimate both the sensitivity and specificity within a range of 5% at α = 0.05 level of significance.

For the second phase, patients with suspected severe malaria were screened from the emergency department at Jinja Regional Referral Hospital, and 180 patients were finally recruited for a randomized controlled trial of a novel adjunctive treatment for severe malaria (nitric oxide). The hypothesis, rationale, and design of the trial, together with inclusion and exclusion criteria are described in detail elsewhere [13,16]. The same three-band RDT (First Response Combo pLDH/HRP2) was used as a screening tool to identify participants with falciparum malaria, and patients were enrolled only if all three bands were positive (i.e., positive HRP2 and pLDH antigenaemia, as well as control band). Trial participants had Giemsa-stained peripheral blood smears (thin and thick) assessed for quantitative malaria parasite density by light microscopy at a College of American Pathologists (CAP) certified, quality-controlled central research laboratory, the Makerere University-John’s Hopkins University (MU-JHU) Core Lab. The positive predictive value of the RDT in this context was calculated, and discordant results (RDT positive, microscopy negative) were examined in further detail. A multiplex polymerase chain reaction (PCR) assay was used to adjudicate discordant results, using methods described previously [17-19].

The accompanying parent or caregiver of all paediatric patients in this study provided full, written, informed consent for participation. Ethical oversight was provided by the Makerere University in Kampala, Uganda and the University Health Network, in Toronto, Canada. The study was conducted in accordance with the declaration of Helsinki and following Good Clinical Practice guidelines.

## Results

For the first phase of the study, 2,000 febrile children were prospectively enrolled from the Emergency Department of the Jinja Regional Referral Hospital, from 15 February 2012 to 11 April 2013. The median (IQR) age was 1.3 (0.75-2.1) years and 886 (45%) were female. Common clinical diagnoses at presentation were malaria, pneumonia, sepsis, meningitis in 1518 (76%), 318 (16%), 220 (11%), and 33 (1.7%) patients, respectively. 65 (3.3%) patients in the cohort had a fatal outcome of their febrile illness.

1,646 children had both a RDT and peripheral blood smear performed. The microscopy result was missing in 284 cases (14%) and the RDT result was missing in 83 (4.2%). Patients with missing data were similar in age and gender to those with available results for both tests: age (median (IQR) 1.4 (0.75-2.0) vs 1.3 (0.75-2.2), p = 0.77) and gender (45% vs 44% female, p = 0.77). HRP2 antigen was detected in 1199 (73%), pLDH in 1033 (63%), and light microscopy was positive in 1031 (63%). Test performance characteristics of the HRP2 line, the pLDH line and the combination of both antigens is given in Table 1. Of note, the specificity of a RDT with all three lines positive (HRP2, pLDH, and control) was 82% (95% CI: 79-85%) compared to 62% (95% CI: 59-66%) for HRP2 alone.

**Table 1:** **Test performance characteristics of RDT bands among febrile hospitalized children (n = 1648) when compared to microscopy**^**1**^

	**Sensitivity**	**Specificity**	**PPV**	**NPV**
**HRP2**	94 (92–95)	62 (59–66)	81 (78–83)	86 (82–89)
**pLDH**	88 (86–90)	80 (77–83)	88 (86–90)	80 (77–83)
**3-bands**^**2**^	88 (85–89)	82 (78–84)	89 (87–91)	80 (76–83)

^1^Numbers of patients within each possible subset of diagnostic test result combinations were as follows: HRP2+, pLDH+, microscopy + 902; HRP2+, pLDH+, microscopy- 113; HRP2+, pLDH-, microscopy + 65; HRP2-, pLDH+, microscopy + 7; HRP2+, pLDH-, microscopy- 119; HRP2-, pLDH+, microscopy- 11; HRP2-, pLDH-, microscopy + 57; HRP2-, pLDH-, microscopy- 374.

^2^Both the HRP2 and pLDH bands (as well as control band) positive.

119 patients (7.2%) had a positive HRP2 line, but negative pLDH and negative peripheral smear. Of these, 72 (61%, p = 0.002 relative to other test results) had received pre-treatment with anti-malarials, suggesting that a significant proportion were false positive HRP2 results. Treatment with an anti-malarial agent prior to presentation was common in the cohort (773 patients, 47%), and was associated with a negative microscopy result (p = 0.001), a negative pLDH result (p < 0.001), but was not statistically significantly associated with a negative HRP2 result (p = 0.09), consistent with persistence of HRP2 antigenaemia in recently treated patients.

For phase 2 of the study, we used the three-band RDT as a screening tool for enrolment of febrile children in a clinical trial conducted at the same centre. 727 patients were screened for eligibility and 180 were enrolled, all of whom had a RDT result with all three lines positive. A CAP quality-assured reference laboratory examined peripheral blood films by light microscopy, and 169 were found to be positive for *P. falciparum* by this method, giving a positive predictive value of 94% (95% CI 89%-97%).

Ten patients had all three RDT lines positive (i.e., HRP2 and pLDH antigenaemia), yet had no parasites visualized on microscopy. One further patient with three positive lines had a diagnosis of *Plasmodium ovale* based on morphologic appearance of parasites under microscopic examination. These samples were tested by PCR for Plasmodium species: all were positive for *P. falciparum*, and none were positive for other Plasmodium species. Further clinical details for these 10 cases are provided in Table 2. Clinical characteristics and laboratory parameters of these 10 patients were consistent with severe malaria, and were not different from the 169 trial participants who had both positive RDT and microscopy. Taken together, these findings suggest that the 10 patients with discordant RDT and microscopy results had severe malaria (false-negative microscopy result), rather than a false-positive RDT result. The patient with *P. ovale* read on blood smear who was positive for *P. falciparum* by PCR also likely had severe malaria from *P. falciparum* and not *P. ovale*.

**Table 2:** **Clinical and prognostic features of microscopy negative (N = 10) and microscopy positive (N = 169) patients enrolled in clinical trial with all three bands positive on RDT**^**1**^

	**Microscopy negative (N = 10)**	**Microscopy positive (N = 169)**	**P value**
Parasite density (geometric mean, range)	0	17,700 (80–696,000)	-
Duration of fever prior to presentation (days), median (IQR)	3.5 (2.0-6.0)	3.0 (2.0-4.0)	0.78
Prostration, n/N (%)	8/10 (80)	155/169 (92)	0.22
Coma, n/N (%)	5/10 (50)	101/169 (60)	0.74
Convulsions, n/N (%)	8/10 (80)	135/169 (80)	1.0
BCS, median (IQR)	3 (2–4)	2 (2–3)	0.33
Deep breathing, n/N (%)	2/10 (20)	84/169 (50)	0.10
LODS^2^, median (IQR)	2 (1–2)	2 (1.5-3)	0.067
pH <7.35^3^, n/N (%)	2/9 (22)	40/137 (29)	1.0
Lactate > 5 mmol/L^3^, n/N (%)	2/10 (20)	57/161 (35)	0.50
Base deficit > 8 mmol/L^3^, n/N (%)	4/9 (44)	65/137 (47)	1.0

^1^One additional patient was diagnosed with *P. ovale* based on microscopy, but PCR identified *P. falciparum* mono-infection.

^2^Lambaréné Organ Dysfunction Score.

^3^pH < 7.35 [20], lactate > 5 mmol/L and base deficit > 8 mmol/L [21] (lactic acidosis) have been identified as markers of poor prognosis in severe malaria.

## Discussion

This study demonstrates that a three-band RDT improves specificity for the diagnosis of falciparum malaria among febrile children hospitalized at a referral hospital in sub-Saharan Africa. The additional specificity provided by the combination of HRP2 and pLDH antigen positivity over HRP2 positivity alone likely relates to the persistence of HRP2 antigen in children recently treated for malaria. This finding was validated in an independent cohort of hospitalized febrile children, using the three-band RDT as a rapid screening tool for a clinical trial, showing that this diagnostic strategy has a high positive predictive value for falciparum malaria.

The sensitivity and specificity of the HPR2 antigen for *P. falciparum* infection in this study were 94% and 62%, respectively. The high sensitivity is consistent with numerous previous studies in various populations, including a meta-analysis of patients screened for uncomplicated malaria (sensitivity = 95%) [5], a systematic review of 48 studies in endemic areas (sensitivity = 93%) [6], and a meta-analysis of over 5,000 non-immune travellers (sensitivity = 95%) [22]. The poor specificity of the HRP2 antigen in this study contrasts with meta-analyses that pool findings from diverse populations (specificity ranges from 95-100%) [5,6,22], but is consistent with observations among febrile hospitalized children in Uganda (specificity of 72% [23] and 85% [24]) and nearby malaria-endemic countries (specificity of 65% in Tanzania [8]). In situations where there is a low probability of recently treated malaria, such as non-immune travellers and outbreak situations in areas of lower endemicity, the specificity of the HRP2 antigen is high (94% to 100%) [15,22].

Typically, three-band RDTs are used in areas where *P. falciparum* and *P. vivax* co-circulate, such as Asia and South America [7]. Beyond their ability to help differentiate vivax from falciparum malaria, the combination of dual antigens on a three-band RDT can point to recently treated falciparum infection because HRP2 and pLDH antigenaemia persists for different periods after eradication of viable parasites. In one report, 4-10% of pLDH tests were still positive compared with 69.7% of the HRP2 tests two weeks after a successfully treated malaria episode [25]. Other studies showed that the mean duration of HRP2 antigenaemia was 32 days [23], and that circulating HRP2 persisted in 68% of patients at 7 days and in 27% at 28 days after treatment [10]. The combination of HRP2 positivity and pLDH negativity may point to recently treated infection, consistent with our findings that a high proportion of patients with this RDT result had received pre-treatment with an anti-malarial agent.

Limitations of this study include the use of an imperfect reference standard: light microscopy of stained peripheral smears. This approach may have led to misclassification of disease state, as illustrated by the second phase of this study, where discordant results adjudicated by PCR showed in all 11 cases that the RDT-based diagnosis (severe falciparum malaria) was likely correct, and the microscopy result likely represented false negative results or incorrect speciation. The first phase of the study employed Field’s stain, which is quicker and less labor intensive than the gold standard Giemsa stain, but has been associated with lower diagnostic sensitivity [26]. Technologists were well trained and experienced, but quality control measures were not in place to test their accuracy and only a single reading was performed. Incomplete testing by either the reference standard or RDT could potentially create sampling bias (1,648/2,000 = 82% patients were tested by both modalities in parallel). The second (validation) phase included only patients with positive three-band RDT result, which allowed us to document the positive predictive value of this diagnostic approach, but did not allow computation of sensitivity, specificity and negative predictive value. The patient population for both phases of the study included children hospitalized for febrile illness in a malaria hyper-endemic zone. While this is arguably the most relevant population in which a specific and rapid diagnostic test is needed (e.g., for inclusion in clinical trials), these results should be extrapolated with caution to other groups, including uncomplicated malaria, non-immune individuals including travelers, and lower incidence environments, where recently treated malaria may be less common.

In summary, the findings of this study indicate that a three-band RDT incorporating both HPR2 and pLDH antigens can improve diagnostic specificity for falciparum malaria over a two-band RDT (HRP2 alone) in a sub-Saharan African context, by excluding false positive HRP2 results due to recently treated infection. Added specificity may be desirable when rapid identification of true cases is required, as for screening patients for inclusion in clinical trials. The three-band RDT has a positive predictive value of 94% in this context.

## Abbreviations

RDT: Rapid diagnostic test; HRP2: Histidine-rich protein 2; CAP: College of American Pathologists; PCR: Polymerase chain reaction; PPV: Positive predictive value; NPV: Negative predictive value; pLDH: Parasite lactate dehydrogenase.

## Competing interests

The authors declare that they have no competing interests.

## Authors’ contributions

MH conceived and designed the study, participated in patient care and data collection, analysed the results, and wrote the manuscript. ALC participated in the study design, supervision of study conduct, and helped to draft the manuscript. ROO participated in study design and logistical planning, and obtained ethical approval from the Ugandan bodies. SN participated in study design and logistical planning. WCL participated in the study design and helped to draft the manuscript. CCJ participated in the study design and helped to draft the manuscript. KCK conceived the study, participated in the study design and helped to draft the manuscript. All authors read and approved the final manuscript.
